# Integration of life cycle assessment and life cycle costing for the eco-design of rubber products

**DOI:** 10.1038/s41598-021-04633-6

**Published:** 2022-01-12

**Authors:** Yahong Dong, Yating Zhao, Hong Wang, Peng Liu, Yan He, Guangyi Lin

**Affiliations:** 1 School of Electromechanical Engineering, Qingdao University of Science and Technology, No. 99 Songling Road, Qingdao, 266061 China; 2 Qingdao Research Center for Green Development and Ecological Environment, Qingdao University of Science and Technology, No. 99 Songling Road, Qingdao, 266061 China

**Keywords:** Engineering, Materials science

## Abstract

Rubber hoses are a category of rubber products that are widely and intensively employed in construction sites for concrete conveying. There has been lack of study to investigate the life cycle environmental and economic impacts of the rubber hoses as an industrial product. In this study, we analyze four types of rubber hoses with the inner layer made of different rubber composites to resist abrasion, i.e., Baseline, S-I, S-II and S-III. Tests of the wear resistance are carried out in the laboratory and S-III shows high abrasion resisting performance with the concrete conveying volume up to 20,000 m^3^ during the service life. Life cycle assessment (LCA) and life cycle costing (LCC) models are established for evaluating the four types of rubber hoses. A target function is developed to integrate LCA and LCC by converting the LCA results to the environmental costs. It is found that S-III can save 13% total cost comparing to Baseline. The production stage is the largest contributor to the environmental single score, while the use stage is the largest contributor to the life cycle cost. Sensitivity analyses are conducted and the results of this study are validated with the previous studies. The integrated method of LCA and LCC developed in this study paves a way for the eco-design of industrial rubber hoses and is potentially applicable to other rubber products.

## Introduction

Rubber products are widely adopted in various applications, including tires, conveyor belts, tubes, wires and cables, shoe soles, hoses, window and door seals, etc. Rubbers are elastomers that can be classified into two types: natural rubber (NR) and synthetic rubber (SR). Natural rubber is isolated from rubber tree (e.g., *Hevea brasiliensis*), while synthetic rubbers are products of chemical syntheses, mainly from petroleum^1,2^. The annual production of rubber per capita is 3.5 kg around the world^3^. The natural rubber production was only 6.8 million tonnes in 2000, and the production grew to 13.6 million tonnes in 2019^4^. Synthetic rubber production in 2000 was 10.9 million tonnes and the amount in 2019 rose to 15.1 million tonnes^5^. Asia dominates the global rubber production^6^, and 92% of the world’s natural rubber is produced in Asia, in particular in China, Thailand, Vietnam and Cambodia^7,8^. Rubber hoses are a category of non-tire rubber products that have been mass-produced. There are various applications for rubber hoses, including transport of gas, liquid or slurry for mining, building construction, engineering machinery, automobiles, aviation, etc. China has the largest number of rubber hose manufacturers in the world^9^, and the annual production exceeded 320 million meters in 2018^10^.

Recent studies of rubber products have focused on the improvement of properties by adding ingredients to the rubber matrix. For example, Xue et al.^11^ added graphene and carbon black to improve the fatigue resistance and the viscoelasticity of natural rubber composites. Lin et al.^12^ added modified aramid fiber to improve the interfacial bonding force between aramid fiber and rubber matrix and consequently extended the services life of the rubber. Sattayanurak et al.^13^ replaced the same amount of silica with a small amount of organic clay, which enhances the Payne effect and improves the traction as well as wear resistance of the tire. Sholeh et al.^14^ studied the nano silica and natural rubber composites with mechanical mixing. The results showed that the appropriate mixing process can improve the dispersion of silica in the rubber matrix, and the rubber can be degraded when the rotor speed reaches 60 rpm. Gao et al.^15^ studied the structure of rubber hoses and observed that the reinforcing ribs have an important influence on the mechanical properties of rubber composite materials. Under an external stress, the reinforcing ribs may result in a greater strength of the rubber hose. Paleri et al.^16^ developed a type of NR composites by a two-stage mixing method, and studied the effect of different types of carbon black in the natural rubber matrix. They found that the addition of mixed fillers based on sustainable biochar (BC) and carbon black (CB) has a significant improvement on the reduction of rolling resistance. This also expanded the application of carbon black in rubber hose. These studies can largely improve the properties of rubber and hence the performance of rubber products. As an urgent need for low-carbon and sustainable development, the evaluation of environmental impacts of rubber products is of great importance. It is necessary to examine whether an improved rubber composite made of new ingredients also have a better environmental performance.

Throughout the life cycle of a rubber product, pollutants are emitted. Particulate matters, sulfur dioxide and nitrogen oxides are generated during the manufacturing processes. Disposal of rubber wastes to landfills may occupy precious land resources and lead to “black pollution”, or even cause fire disaster without proper treatment^17,18^. To evaluate the environmental performance of rubber products, life cycle assessment (LCA) has been adopted as an effective instrument. The previous LCA studies of rubbers include the evaluation of greenhouse gas emissions^19^, the calculation of water footprint^20^, the evaluation of environmental performance of latex^21^ and rubber ^22^, the environmental impacts of life cycle of tires^23–25^, LCA of scrap tires^26–28^, etc.

To understand whether the improvements of rubber properties are economically efficient, the cost to produce rubber materials and manufacture final rubber products should be accounted for. As a counterpart of LCA, the life cycle costing (LCC) is a method to evaluate all costs of a product throughout the whole life cycle stages^29^. However, there are only a few studies related to LCC of rubber products. For example, Kang et al.^30^ conducted both LCC and LCA on a new generation wide-base tire and proved significant savings in life cycle energy consumption and cost of the product.

Although the previous studies have paid attention to the environmental impacts and economic costs of rubber products, there are a number of shortages. It is found that tires have been intensively studied^31^, whereas the non-tire rubber products, such as rubber hoses, shoes, wires, etc. were rarely studied. Several issues are still not clear, such as the emission range of rubber products, the hotspots of materials/processes, the life cycle costs, etc. Most importantly, it is unknown whether the improvement of properties of rubber also leads to environmental and economic savings for the non-tire rubber products. The case studies of different types of rubber products are thus needed.

Rubber hoses are a category of rubber products that are intensively employed in many construction sites for concrete conveying. Normally several meters long and decimeters in diameter, a rubber hose is essential for pumping the ready mixed concrete to the planned area, due to its good flexibility. In this study, a comprehensive analysis of LCA and LCC is conducted for four types of rubber hoses each with different rubber composites that were developed in the laboratory. LCA is carried out to evaluate 18 midpoint impact categories and 3 endpoint damage categories. The LCA results are subsequently converted to monetary values and integrated with LCC results. Sensitivity analyses are conducted on the ingredients, materials, processes and resources to detect hotspots. The integrated method of LCA and LCC can promote the transformation of traditional rubber industry towards a green rubber industry and provide a solid methodological framework of eco-design for rubber products.

## Materials and methods

### Description of the rubber hoses

**Figure 1:**
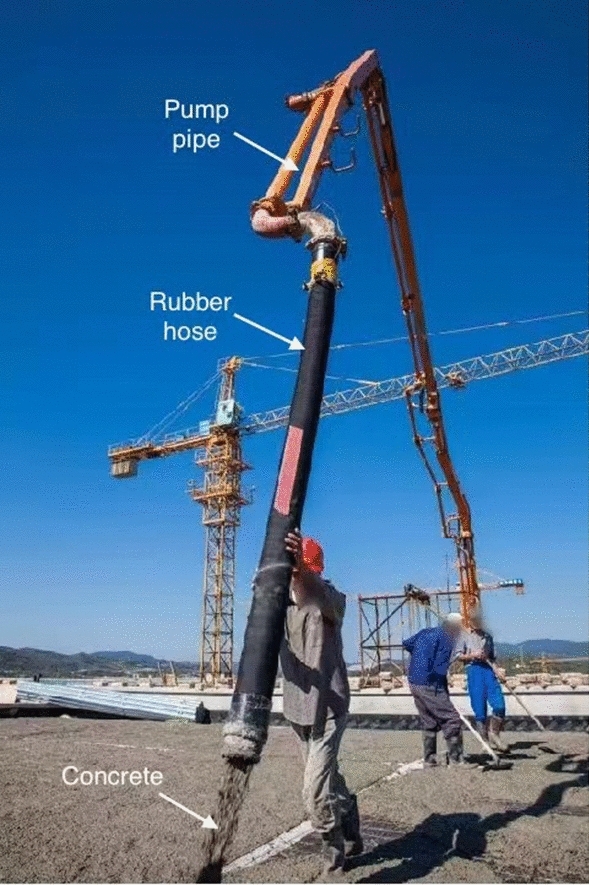
Rubber hose being used for concrete pumping on a construction site in Qingdao, China (Photo source: the manufacturer of the studied rubber hoses).

**Figure 2:**
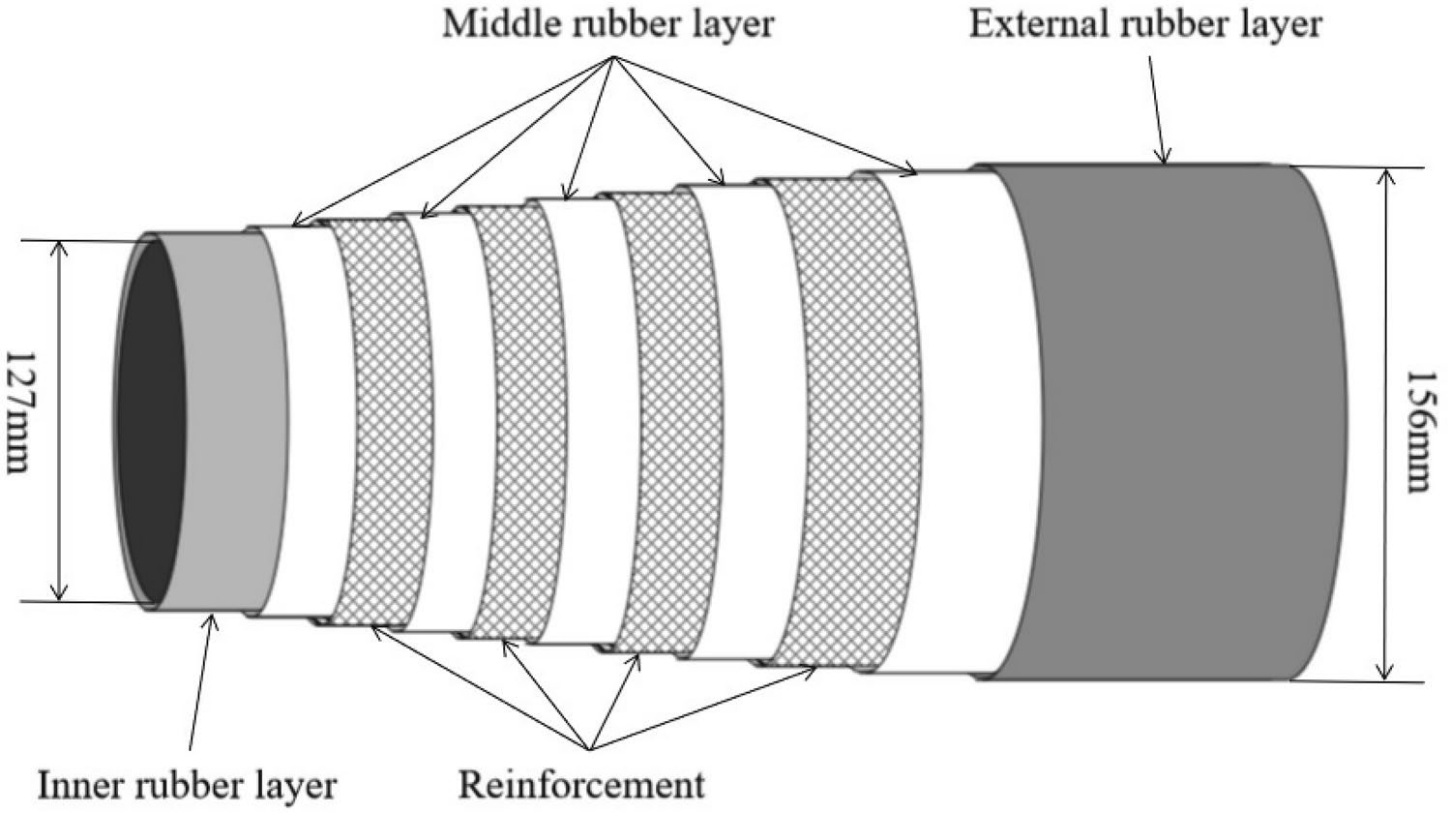
Schematic illustration of the rubber hose composition.

The rubber hoses are used on construction site for concrete conveying (Fig. 1). A rubber hose is connected to the concrete pumping pipe which is installed along the robotic arm of a boom pump. Ready-mix concrete is delivered through the pumping pipe and screeded accurately and quickly through the rubber hose for concrete placement. The studied rubber hoses consist of inner rubber layer, middle rubber layer, reinforcement, and an external rubber layer (Fig. 2). The inner rubber layer is compounded of natural rubber (NR, *cis*-1,4 polyisoprene), polybutadiene rubber (BR, *cis*-1,4 polybutadiene), carbon black, tackifier resin, etc. The middle rubber layer is compounded of chloroprene rubber (CR), BR, carbon black, silica sand, aromatic oil, etc. The reinforcement is made of steel wire to improve mechanical properties of rubber hose. The external rubber layer is compounded of styrene butadiene rubber (SBR), BR, carbon black, tackifier resin, etc. The inner diameter of the rubber hoses is 127 mm and the outer diameter is 156 mm. The length of the rubber hoses is 3 m.

**Figure 3:**
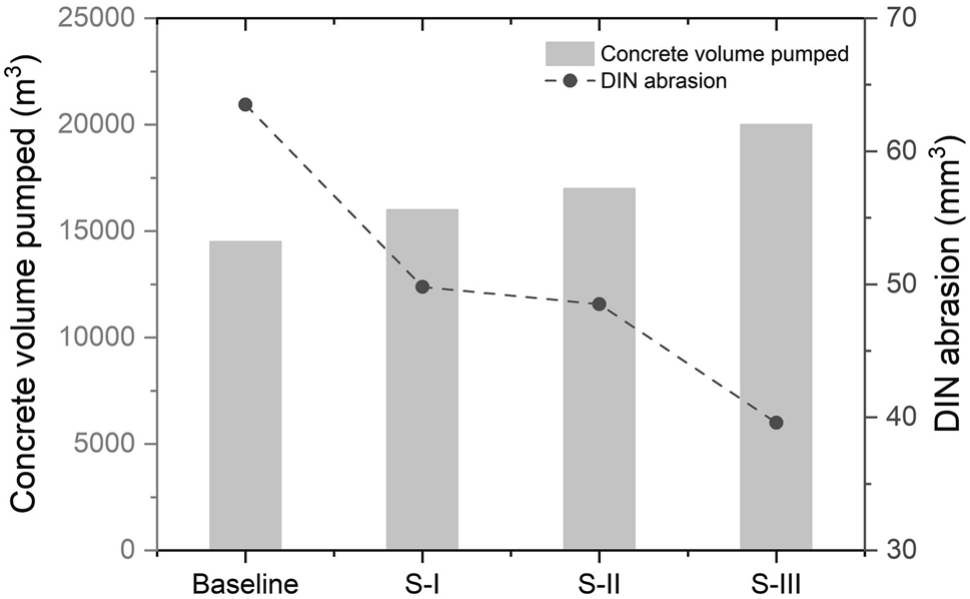
Laboratory tests of DIN abrasion and volume of concrete pumping during the service life of rubber hoses.

As boom pumps are usually adopted in construction sites, large volume of concrete can be delivered. The rubber hose should have a high wear resistance in its inner rubber layer. It is a critical factor that determines the service life of a rubber hose. In the laboratory, experiments are carried out to examine the DIN abrasion of four types of rubber hoses with different rubber composites for the inner rubber layer. The composite material formulas of the four rubber composites are provided in Table 1. The experiment results of DIN abrasion and the estimated service life of the four composites are shown in Fig. 3. The Baseline is the traditional composite which has been used as the inner layer rubber for rubber hoses. The DIN abrasion of Baseline is tested as 63.5 mm^3^, which is the largest value among the four composites. Consequently, the concrete volume conveyed during the service life of a rubber hose with the Baseline is the smallest (14,500 m^3^). To improve the performance of the inner rubber layer, another three composites were designed and tested. It is found that S-III has the best performance with DIN abrasion of 39.6 mm^3^ and the conveyed concrete volume can be up to 20,000 m^3^. S-III is now being used in producing high wear-resistant rubber hoses in a factory.

**Table 1:** Material formula of four rubber composites (in parts per hundred of rubber, phr).

Sample	Baseline	S-I	S-II	S-III
NR	30	30	60	40
BR	70	70	40	60
Sulfur	1.5	1.5	1.5	1.5
Accelerator	1.5	1.5	1.5	1.5
CZ	1	0	1.5	1
DM	0.5	0.5	0	0.5
NS	0	1	0	0
Zinc oxide	5	5	5	5
Stearic acid	1	1	1	1
Carbon black	55	55	55	55
Type of carbon black	N234	N330	N234	N110
Tackifier resin	8	8	8	8
Antiager	2	2	2	2
Paraffin	1	1	1	1
White carbon black	10	0	0	0
Silane coupling agent	4	0	0	0

CZ, DM, NS—accelerator; phr—the quantity of additives per 100 units of base polymer.

### System boundary and functional unit

**Figure 4:**
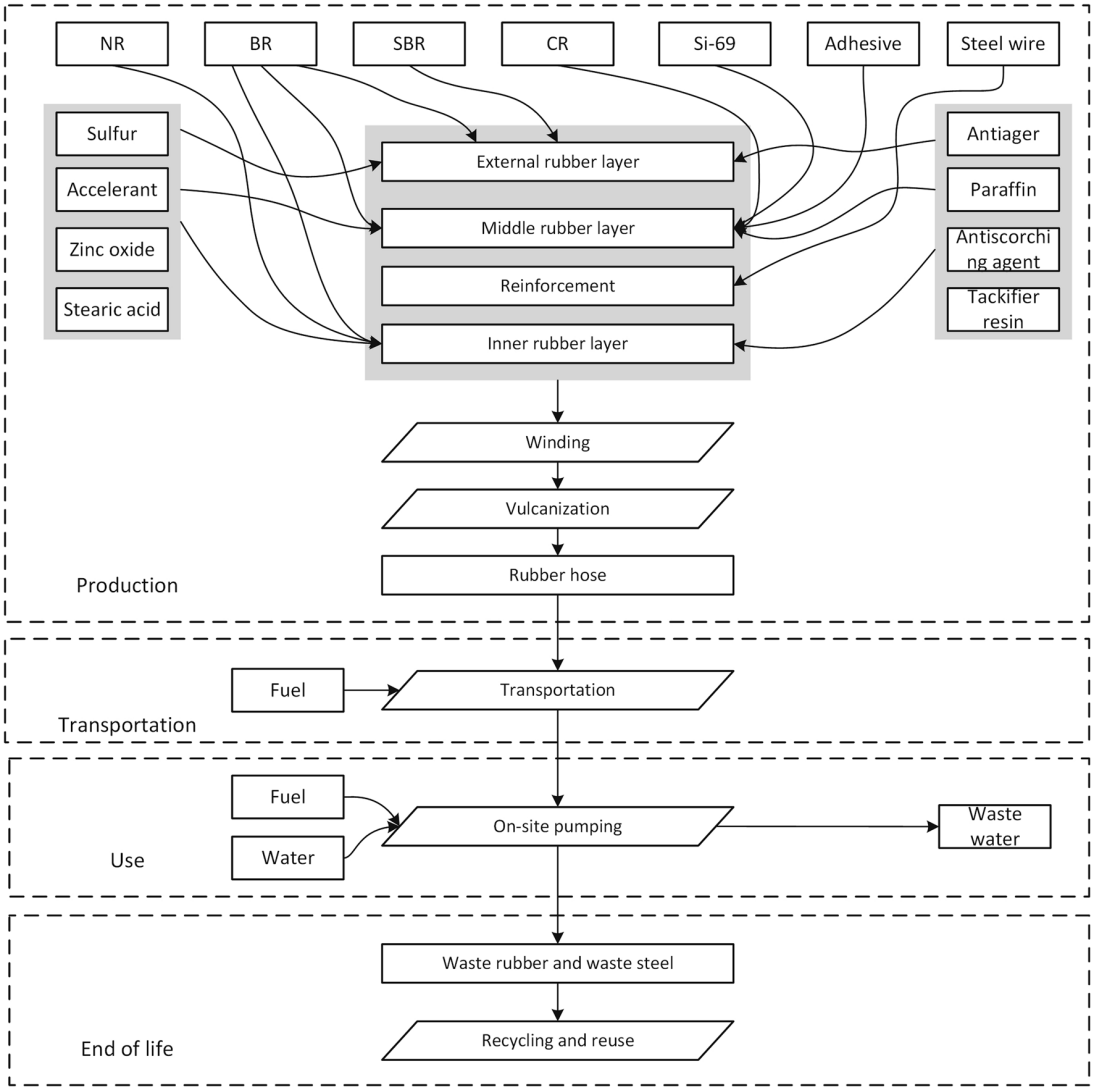
System boundary of the LCA and LCC models.

The system consists of the entire life cycle stages of a rubber hose, including production, transportation, use and the end-of-life recycling and reuse (Fig. 4). In the production stage, the materials, transportation of materials to factory, and manufacturing of rubber hoses are included. The in-factory processes include winding rubber layers and reinforcement, vulcanization, and storage. The manufactured rubber hoses are then transported to a construction firm and placed in the storage. In the next step, a rubber hose is installed into a boom pump and the boom pump truck is driven to the construction site for concrete pumping. During the use stage, the rubber hoses are flushed by water every 200 m^3^ concrete pumped (as consulted with the project manager). Wastewater is reused with proper treatment in a sedimentation tank. There are two types of waste materials generated in the end-of-life stage, viz*.* waste rubber and steel scrap, and both are recycled. All the processes within the system boundary are analyzed and missing information due to data unavailability should be less than 1% (cut-off criteria) of the total environmental impact.

The functional unit (FU) is a reference to which the inputs and outputs of the studied product are normalized. According to Dong et al.^31^, 76% LCA studies of rubber products (e.g., tires) used mass-based and number-based FUs. Following the common practice of LCA of rubber products, the FU is one rubber hose (as shown in Figs. 1 and 2). It is a rubber hose of 164 kg in weight and 3 m in length, with an inner diameter of 127 mm and an outer diameter of 156 mm.

### Data inputs

#### Inputs of the LCA model

The four rubber hoses in this study are all manufactured by a factory in Ningbo and transported to a construction site in Qingdao for use. After the end of their service life, they will be recycled in Qingdao.

The input data in LCA model are mainly obtained from the questionnaire survey, and factory manager and construction worker are the questionnaire fillers of this study. The factory manager mainly provides data on the production and transportation stages of the rubber hoses, while the construction worker mainly provides data related to the use and end-of-life stages of the rubber hoses. Data that could not be collected by questionnaire survey (i.e., beyond the scope of management of the person who filled in the questionnaire) were supplemented by the data in previous research and national standard. The data sources are described in detail in this section.

##### Production stage

The input data in the production stage were mainly acquired from a questionnaire survey to the factory manager in September 2020. The questionnaire is attached as the Supplementary Material. After received the feedback, we conducted several rounds of interviews with the factory manager to verify the quality of the data. The input data of the inner rubber layer for the four types of rubber hoses are shown in Table 2 and for the middle and external rubber layers the data are shown in Table 3. The upstream datasets are obtained from the Ecoinvent database and literature. The weight of steel wire (reinforcement) is 60 kg and the upstream dataset is from the Ecoinvent database. The weight of a rubber hose is 164 kg with 60 kg of inner rubber layer, 26.4 kg of middle rubber layer, 60 kg of reinforcement and 17.6 kg of external rubber layer. Delivery of raw materials to the rubber factor is involved in the LCA model. As shown in Table 4, the origins of the suppliers are provided, and the distances are calculated using the online Baidu map. In the LCA model, the 7.6–16 t truck is selected according to the consult with the factory manager. The information of electricity consumption is collected through the questionnaire, while the direct emissions during the manufacturing process are estimated based on GB27632-2011: Emission standard of pollutants for rubber products industry (Table 5).

**Table 2:** Input data of the inner rubber layer (FU: one rubber hose).

Ingredient	Baseline (g)	S-I (g)	S-II (g)	S-III (g)	Data source	Upstream datasets*
NR	9523.8	10,285.7	20,571.6	13,714.2	Questionnaire	^24^
BR	22,222.2	24,000	13,714.2	20,571.6	Questionnaire	Ecoinvent
Carbon black N110	0	0	0	18,857.4	Questionnaire	Ecoinvent
Carbon black N234	17,460.3	0	18,857.4	0	Questionnaire	Ecoinvent
Carbon black N330	0	18,857.16	0	0	Questionnaire	Ecoinvent
Accelerator CZ	317.4	0	514.2	342.6	Questionnaire	^32^
Accelerator DM	158.7	171.4	0	171.6	Questionnaire	^32^
Accelerator NS	0	342.8	0	0	Questionnaire	^33^
Zinc oxide	1587.3	1714.2	1714.2	1714.2	Questionnaire	Ecoinvent
Stearic acid	317.4	342.8	342.6	342.6	Questionnaire	Ecoinvent
Sulfur	476.2	514.2	514.2	514.2	Questionnaire	Ecoinvent
Antiager 4020	634.9	685.7	685.8	685.8	Questionnaire	^34^
Tackifier resin	2539.6	2742.8	2742.6	2742.6	Questionnaire	^35^
Paraffin	317.4	342.8	342.6	342.6	Questionnaire	Ecoinvent
SI-69	1269.8	0	0	0	Questionnaire	^36^
White carbon black	3174.6	0	0	0	Questionnaire	Ecoinvent

*Upstream data source refers to the database for the upstream datasets or the literature from which the upstream data are obtained.

**Table 3:** Input data of the middle and external rubber layers (FU: one rubber hose).

Ingredient	Middle (g)	External (g)	Data source	Upstream dataset*
BR	5035.0	2731.6	Questionnaire	Ecoinvent
CR	11,748.0	0	Questionnaire	^37^
SBR	0	7024.3	Questionnaire	^38^
Carbon black N234	0	5853.5	Questionnaire	Ecoinvent
Carbon black N330	3356.7	0	Questionnaire	Ecoinvent
Accelerator DM	167.9	48.7	Questionnaire	^32^
Accelerator NS	0	175.6	Questionnaire	^33^
Zinc oxide	839.2	487.8	Questionnaire	Ecoinvent
Magnesium oxide	671.3	0	Questionnaire	Ecoinvent
Stearic acid	167.9	97.5	Questionnaire	Ecoinvent
Sulfur	134.3	97.5	Questionnaire	Ecoinvent
Antiager 4010NA	251.8	195.1	Questionnaire	^34^
Tackifier resin	0	780.5	Questionnaire	^35^
Anti-scorching agent CTP	0	9.6	Questionnaire	^32^
Aromatic oil	1678.2	0	Questionnaire	Ecoinvent
Adhesive	167.9	0	Questionnaire	^39^
Paraffin	0	97.5	Questionnaire	Ecoinvent
SI-69	503.4	0	Questionnaire	^36^
White carbon black	1678.2	0	Questionnaire	Ecoinvent

*Upstream data source refers to the database for the upstream datasets or the literature from which the upstream data are obtained.

**Table 4:** Transportation distances of raw materials from suppliers to the factory (in Ningbo, China).

Material	Distance (km)*	Location of supplier
NR	1340	Dongguan, China
BR	1365	Beijing, China
CR	1340	Dongguan, China
SBR	1340	Dongguan, China
Carbon black N110	1004	Dongying, China
Carbon black N234	1004	Dongying, China
Carbon black N330	1004	Dongying, China
Accelerator CZ	1350	Guangzhou, China
Accelerator DM	1350	Guangzhou, China
Accelerator NS	1350	Guangzhou, China
Zinc oxide	383	Jiangsu, China
Magnesium oxide	1350	Guangzhou, China
Stearic acid	434	Nanjing, China
Sulfur	476	Yancheng, China
Antiager 4010NA	317	Changzhou, China
Antiager 4020	317	Changzhou, China
Tackifier resin	1098	Puyang, China
Anti-scorching agent CTP	1350	Guangzhou, China
Aromatic oil	1350	Guangzhou, China
Adhesive	302	Jiangsu, China
Paraffin	1203	Anyang, China
SI-69	1350	Guangzhou, China
White carbon black	1350	Guangzhou, China
Steel wire	317	Changzhou, China

*Distances of transportation of ingredients are estimated using Baidu Map (map.baidu.com).

**Table 5:** Emissions and electricity consumption during manufacturing (FU: one rubber hose).

Category	Item	Amount*	Unit
Emissions	Chemical oxygen demand	2.98E−02	kg
	Nitrogen, total	4.26E−03	kg
	Phosphorus, total	2.13E−04	kg
	Particulates	1.58E−03	kg
Consumption	Electricity	67	kWh

*Emissions are estimated based on GB27632-2011; the amount of electricity is obtained by questionnaire survey to the manufacturer.

##### Transportation stage

The transportation of rubber hoses to the construction site is included in the system boundary. The construction site is located in Qingdao, China, and the distance from Ningbo to Qingdao is 893 km (calculated using the Baidu map). As consulted with the factory manager, rubber hoses are delivered by the 12 t truck with the capacity of 73 rubber hoses. The dataset of “Transport, freight, lorry 7.5–16 metric ton, Euro 6 RoW market for transport” in Ecoinvent is selected to model the transportation of rubber hoses. Distance and weight are normalized to 1 rubber hose.

##### Use stage

The concrete pumping capacity of the four types of rubber hoses are: 14,500 m^3^ (Baseline), 16,000 m^3^ (S-I), 17,000 m^3^ (S-II) and 20,000 m^3^ (S-III). Diesel consumption is obtained from the questionnaire (Table 6). Water consumption takes place in removing the concrete retained in the pump pipe. As consulted with construction workers, 8 m^3^ water is needed to clean up the pump pipe (61 m). On average, after pumping 200 m^3^ concrete, the pipe should be cleaned. The water consumption for each rubber hose is then estimated based on their pumping capacity (Table 6). During the use phase, 70% of inner rubber layer is lost due to the abrasion during concrete pumping. The crumb rubber is mixed with the concrete but not emitted to the environment.

**Table 6:** Inputs for the use stage of one rubber hose.

Item	Baseline	S-I	S-II	S-III	Unit	Source
Diesel	339	374	398	468	kg	Questionnaire
Water	28.52	31.48	33.44	39.34	m^3^	^40^
Wastewater	28.52	31.48	33.44	39.34	m^3^	^40^

##### EoL stage

The waste rubber is recycled to produce the reclaimed rubber^41^, and the inputs for the end-of-life stage of one rubber hose are provided in Table 7. As 70% of rubber is lost due to abrasion, only 30% becomes the waste rubber and all of the waste rubber is recycled. The reclaimed rubber can replace 50% virgin rubber to make synthetic rubber. The waste treatment of rubber hoses is together with other rubber products, such as tires and belts. The electricity, water and diesel consumptions of waste rubber as well as the emissions are estimated according to previous study^42^. Transportation of waste to the recycling factory is by a 3.3 t truck and the distance is 50 km. The steel wire in the reinforcement is 95% recycled (57 kg) whereas 5% (3 kg) is disposed as solid waste.

**Table 7:** Inputs for the end-of-life of one rubber hose.

Category	Item	Amount	Unit	Source
Consumption	Electricity	106	kWh	^42^
	Water	0.978	m^3^	^42^
	Diesel	1.38	kg	^42^
Emissions	SO_2_	0.0992	kg	^42^
	NO_X_	0.0523	kg	^42^
	H_2_S	0.0248	kg	^42^
	Particulates	0.0482	kg	^42^
Transportation	Rubber	50	km	Questionnaire
	Steel	50	km	Questionnaire
Recycled material	Recycled rubber	31.2	kg	Questionnaire
	Recycled steel	57	kg	^42^
Waste	Waste wire	3	kg	^42^

#### Inputs of the LCC model

The inputs of the LCC model are given in Table 8. The prices of materials at the production stage are collected from the Alibaba website and then verified with the factory manager. The electricity price in Ningbo and Qingdao is obtained from the government website in December 2020. Water price is also obtained from the government website in December 2020. Price of diesel is collected from the local gas station in September 2020. Transportation costs are calculated based on the distance and truck capacity. To deliver the rubber hoses from Ningbo to Qingdao, 12 t truck is used (after consulting with the factory manager), and the total cost per trip (893 km) is 5010 CNY and 69 CNY per one rubber hose. The end-of-life waste is delivered by a trash truck with 3.3 t and 3.6 m height. The price per trip is 410 CNY and on average it is 11 CNY per rubber hose. The prices of waste steel and reclaimed rubber are obtained from the construction project manager.

**Table 8:** LCC inputs of the four life cycle stages (data source: the Alibaba website).

LC stages	Item	Price	Units
Production	NR	11,760	¥/t
	CR	32,000	¥/t
	SBR	9500	¥/t
	BR	11,300	¥/t
	Sulfur	8000	¥/t
	Accelerator CZ	22,000	¥/t
	Accelerator DM	16,800	¥/t
	Accelerator NS	27,500	¥/t
	Zinc oxide	18,600	¥/t
	Stearic acid	8600	¥/t
	Carbon black	6800	¥/t
	Tackifying resin	11,400	¥/t
	Antiager	19,400	¥/t
	Paraffin	7800	¥/t
	Anti-scorching agent	27,500	¥/t
	Magnesium oxide	6800	¥/t
	Adhesive	15,500	¥/t
	Silica	5800	¥/t
	Silane coupling agent	26,000	¥/t
	Aromatic oil	5200	¥/t
	Steel wire	3700	¥/t
	Electricity (production) Ningbo	0.6644	¥/kWh
Transportation	Transport (Ningbo to Qingdao)	5010	¥
Use stage	Water (use stage)	5.4	¥/m^3^
	Diesel (use stage)	5.3	¥/L
End-of-life	Transport (waste)	410	¥
	Reclaimed rubber	− 3000	¥/t
	Waste steel wire	− 2750	¥/t
	Electricity (end-of-life) Qingdao	0.725	¥/kWh
	Diesel (end-of-life)	5.3	¥/L

¥ is Chinese Yuan (CNY).

### Impact assessments

#### Impact assessments of the LCA model

In this study, ReCiPe 2016 is adopted as the life cycle impact assessment (LCIA) method, since it is a harmonized method that provides both midpoint and endpoint assessments^43^. The midpoint approach of ReCiPe 2016 encompasses 18 impact categories, while the endpoint approach provides three damage categories (referring to Table 9). There are three different perspectives of ReCiPe 2016, viz*.,* Individualist (I), Hierarchist (H), and Egalitarian (E). Individualist is the short-term model (e.g., 20 years of climate change impact), Hierarchist is the balance perspective (e.g., 100 years of climate change impact), and Egalitarian is the long-term model (e.g., 500 years of climate change impact). Hierarchist perspective is the most adopted in LCA studies. We adopt ReCiPe 2016 Midpoint (H) for the midpoint assessment and ReCiPe 2016 Endpoint (H/A) for the endpoint assessment. In the endpoint approach, “A” refers to the average weighting set. The LCA model is established in SimaPro, a widely used commercial software developed by PRé.

**Table 9:** Environmental impacts of the four types of rubber hoses.

Approach	Impact category	Abbr	Unit	Baseline	S-I	Diff (%)	S-II	Diff (%)	S-III	Diff (%)
Midpoint	Climate change	CC	kg CO_2_ eq	8.47E+02	8.64E+02	2.04	*8.46E*+*02*	− 0.18	**9.04E**+**02**	6.67
	Ozone depletion	OD	kg CFC-11 eq	*6.48E−04*	6.60E−04	1.90	7.23E−04	11.61	**7.74E−04**	19.54
	Ionizing radiation	IR	kBq Co-60 eq	*3.19E*+*01*	3.28E+01	2.90	3.39E+01	6.26	**3.60E**+**01**	12.99
	Photochemical oxidant formation: human health	HOF	kg NOx eq	*2.41E*+*00*	2.42E+00	0.59	2.50E+00	3.85	**2.67E**+**00**	11.03
	Particulate matter formation	PMF	kg PM2.5 eq	1.70E+00	*1.66E*+*00*	− 2.15	1.85E+00	9.06	**1.90E**+**00**	11.90
	Photochemical oxidant formation: terrestrial ecosystems	EOF	kg NOx eq	*2.57E*+*00*	2.59E+00	0.73	2.67E+00	3.59	**2.85E**+**00**	10.90
	Terrestrial acidification	TA	kg SO_2_ eq	4.19E+00	*4.01E*+*00*	− 4.14	4.51E+00	7.81	**4.72E**+**00**	12.82
	Freshwater eutrophication	EF	kg P eq	1.74E−01	*1.69E−01*	− 2.81	**1.84E−01**	5.59	1.81E−01	4.02
	Marine eutrophication	ME	kg N eq	1.73E−02	*1.73E−02*	− 0.09	**1.78E−02**	3.14	1.78E−02	3.05
	Terrestrial ecotoxicity	TET	kg 1,4-DCB	*2.48E*+*03*	2.53E+03	2.21	2.59E+03	4.65	**2.70E**+**03**	9.06
	Freshwater ecotoxicity	FET	kg 1,4-DCB	1.98E+01	*1.74E*+*01*	− 12.15	**2.20E**+**01**	11.23	2.11E+01	6.33
	Marine ecotoxicity	MET	kg 1,4-DCB	2.75E+01	*2.46E*+*01*	− 10.68	**3.05E**+**01**	10.81	2.95E+01	7.03
	Human toxicity: cancer	HTc	kg 1,4-DCB	3.29E+01	*3.27E*+*01*	− 0.51	3.35E+01	1.83	**3.43E**+**01**	4.18
	Human toxicity: non-cancer	HTnc	kg 1,4-DCB	6.04E+02	*5.01E*+*02*	− 17.09	**6.88E**+**02**	13.82	6.40E+02	5.96
	Agricultural land occupation	ALO	m^2^ a crop eq	1.46E+01	*1.45E*+*01*	− 1.01	1.48E+01	1.46	**1.52E**+**01**	3.84
	Mineral resource depletion	MRD	kg Cu eq	1.03E+00	*3.82E−01*	− 62.96	**1.55E**+**00**	50.66	1.24E+00	20.47
	Fossil resource depletion	FRD	kg oil eq	*6.32E*+*02*	6.76E+02	6.95	6.86E+02	8.56	**7.80E**+**02**	23.42
	Water depletion	WD	m^3^	*3.45E*+*01*	3.74E+01	8.56	3.94E+01	14.50	**4.54E**+**01**	31.76
Endpoint	Human health		Pt	3.59E+01	*3.54E*+*01*	− 1.35	3.79E+01	5.64	**3.93E**+**01**	9.42
	Ecosystems		Pt	*2.26E*+*00*	2.27E+00	0.54	2.33E+00	2.75	**2.47E**+**00**	9.17
	Resources		Pt	*1.79E*+*00*	1.93E+00	7.95	1.97E+00	9.93	**2.27E**+**00**	26.60
	Single score		Pt	3.99E+01	*3.96E*+*01*	− 0.82	4.22E+01	5.67	**4.40E**+**01**	10.18

Bolded values refers to the highest environmental impacts among the four rubber hoses; Italicised values refers to the lowest environmental impacts among the four rubber hoses. “Diff.” refers to the difference between the rubber hose with the baseline rubber hose.

#### Impact assessments of the LCC model

The indicator in the life cycle costing (LCC) model is a monetary value. In this study, Chinese Yuan (CNY, ¥) is adopted as the metric of the indicator. The results of other currencies are converted to CNY according to the exchange rates, e.g., 6.536 CNY/USD^44^. As the whole life cycle of a rubber hose from production to EoL is within 2 years (usually within 1 year), inflation, fluctuation and interest rates are not considered in the LCC model. The external costs due to environmental impacts are included through an objective function as described in the next subsection.

### Integration of LCA and LCC

To integrate the LCA and LCC, the environmental impacts are monetarized through the conversion factors of damage categories. The costs due to environment impacts can be calculated by:

1 $$C_{LCA} = \mathop \sum \limits_{i = 1}^{3} m_{i} ED_{i} ,$$

where *m*_*i*_ is the conversion factor of the *i*th damage category, *ED*_*i*_ is the environmental assessment results of the *i*th damage category of LCIA.

There are three damage categories in the ReCiPe 2016 Endpoint, namely human health (disability-adjusted life years, DALYs), ecosystem (lost species-year) and resource depletion (US dollars). Several conversion factors are available for both human health and ecosystem^45^. As given in Ögmundarson et al.^46^ the conversion factors for human health and ecosystem are 100,000 USD/DALY and 65,000 USD/species.yr, respectively, which are adopted in this study. The influence of the conversion factors of monetary values is further discussed through a sensitivity analysis.

The monetary result of LCA can then be integrated with LCC through the multi-objective optimization method. The objective function is given as:

2 $$F_{C} = C_{LCA} + C_{LCC} ,$$

where $F_{C}$ is the total cost, from both traditional life cycle costs and the external environmental costs; *C*_*LCA*_ is the monetary value of environmental assessment results; *C*_*LCC*_ is the economic cost of the whole life cycle of a rubber hose. The $F_{C}$ is calculated for the four types of rubber hoses and the one with least total cost is considered as the optimized option.

### Ethics approval

The paper is original. No other ethical issues.

### Consent to participate

We declare that all the authors were informed before submission and agree to publish the paper.

### Consent for publication

We declare that all the authors agreed to publish the paper in this journal.

## Results

### Results of life cycle assessment (LCA)

#### Midpoint and endpoint results

The midpoint and endpoint results of the four rubber hoses are given in Table 9. For the impact categories of OD, IR, HOF, EOF, TET, FRD and WD, the type Baseline has the lowest environmental impacts, while S-III has the largest impacts. This is mainly caused by the longer service life of S-III as compared to the Baseline, which increases the environmental impacts. On the other hand, the environmental impacts of the remaining 11 impacts categories are not correlated to the service life of rubber hoses, but mainly affected by the composites of inner rubber layer.

In the impact categories of EF, ME, FET, MET, HTnc, and MRD, S-I has better performance than Baseline, and S-II has greater environmental impacts than S-III. The environmental impacts caused by the four rubber hoses are ranked as S-I, Baseline, S-III and S-II, from small to large. This is caused by the amount of Accelerator CZ. As shown in Table 10, the amount of Accelerator CZ is the largest in S-II but the lowest in S-I, corresponding to the environmental impacts of the rubber hoses in these impact categories. For example, MRD has the contribution of Accelerator CZ to the production stage: 0% of S-I, 10.7% of Baseline, 11.4% of S-III, and 16.2% of S-II. By examining the upstream processes of Accelerator CZ, it is found that metal catalysts are used in Accelerator CZ, leading to large environmental impacts in these impact categories.

**Table 10:** The accelerators used in the four types of rubber hoses.

Type	Baseline	S-I	S-II	S-III	Unit
Accelerator NS	175.648	518.488	175.648	175.648	g
Accelerator DM	375.416	388.076	216.656	388.256	g
Accelerator CZ	317.460	0	514.200	342.600	g

For the impact categories of PMF, TA, HTc and ALO, S-III has the largest environmental impacts, and S-I has the lowest impacts. This is jointly caused by the different amount of Accelerator CZ and the different fuel consumptions. S-I is the only rubber hose with no Accelerator CZ, thus the environmental impacts of S-I is the smallest. On the other hand, the fuel consumption is larger in S-III than in S-II, leading to large environmental impacts of S-III.

The impact category of CC demonstrates different patterns. It is found that S-III has the largest emission of 851 kg CO_2_ eq, whereas S-II has the lowest emission of 793 kg CO_2_ eq. This is primarily caused by the ratio of NR and BR in the inner rubber hose layer. The NR:BR ratio of S-II is 6:4, and this is 3:7 in Baseline, resulting in lower carbon emissions of S-II than Baseline.

With respect to the endpoint results, it is found that S-III has the largest impacts to all the three damage categories. S-I has the lowest impact to human health. Baseline has the lowest impacts to ecosystem and resources. S-III has the largest single score, while S-II has the smallest single score.

#### Contributions of impact categories to the single score

**Figure 5:**
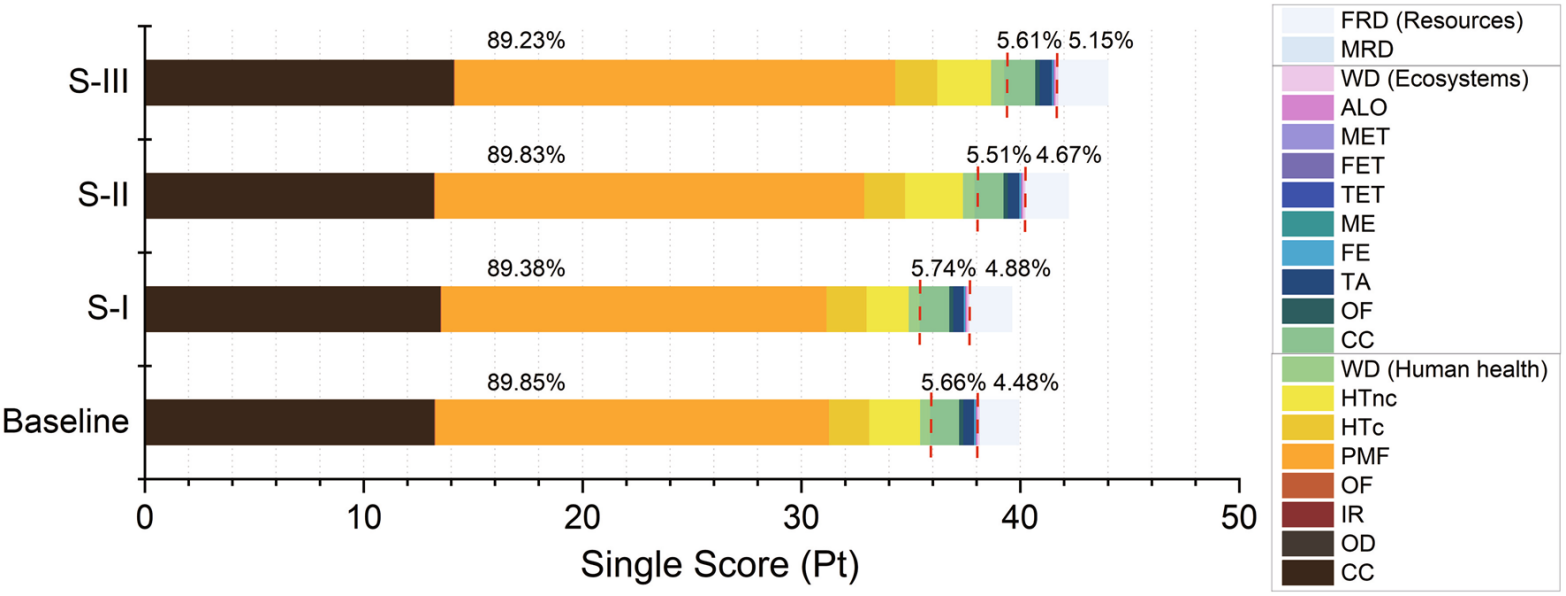
Contributions from impact categories towards the single score.

Figure 5 gives the contribution analysis of the impact categories based on the single score of the endpoint approach. It is found that human health has the most contribution (89.23–89.85%) to the single score, while ecosystems (about 5.51–5.74%) and resources (4.48–5.15%) have apparently lower contributions. For the individual impact categories, the most contributive impact category is PMF, which has impact of 17.6–20.1 Pt, accounting for approximately 45% of the single score. The second largest contributor is CC (Human health), which has impact of 13.2–14.1 Pt. CC (Human health) and CC (Ecosystems) can jointly account for 34.4–37.5% of the single score. Other large contributors include HTc, HTnc, WD (Human health), and FRD.

#### Contributions of materials and processes to the single score

**Figure 6:**
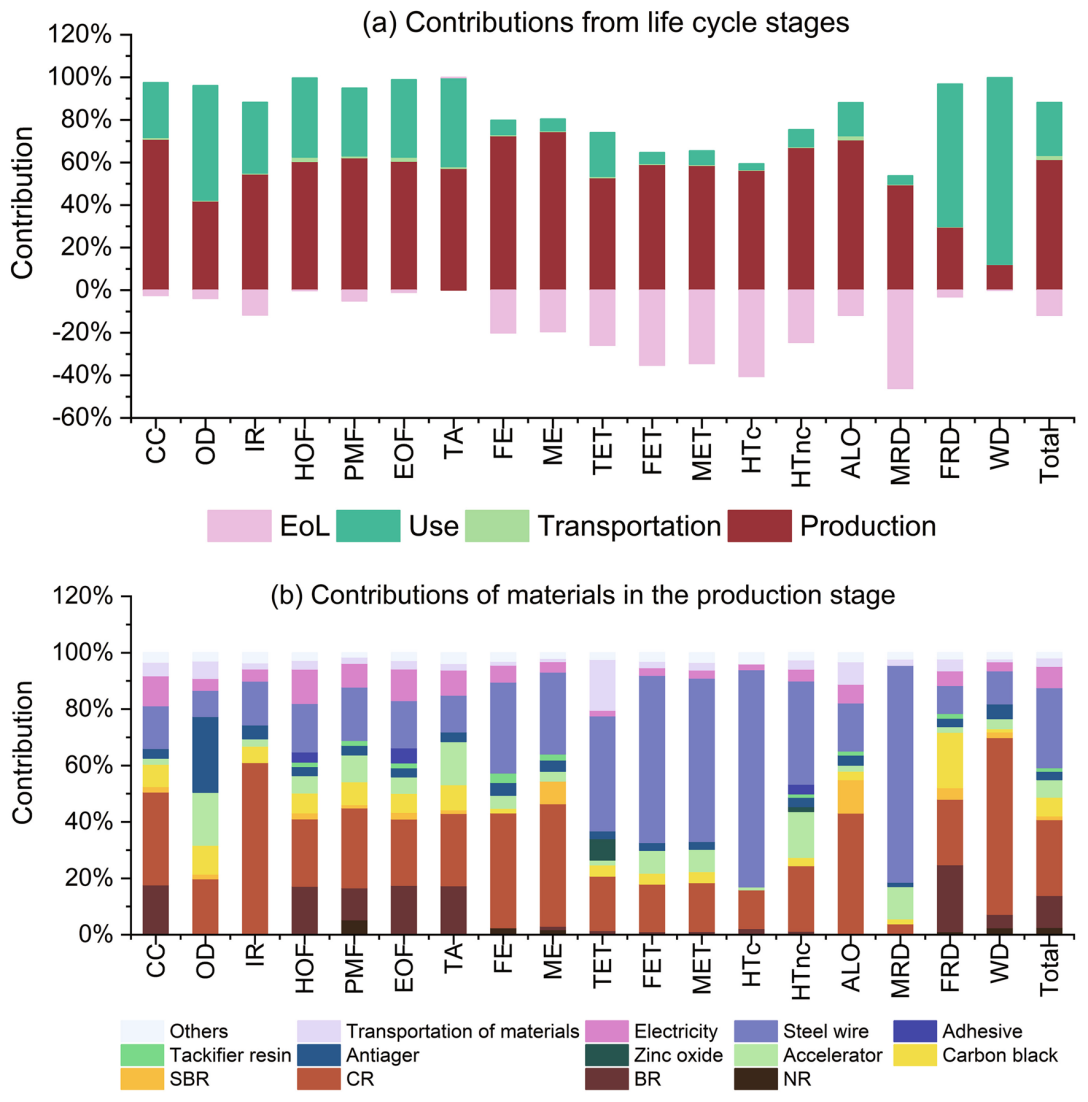
Contribution analysis of the high wear resistant rubber hose (S-III). (**a**) Contributions of life cycle stages; and (**b**) contributions of materials and processes in the production stage. Note: “total” indicates environmental single score; “others” in (**b**) refer to raw materials with contributions less than 1%.

Figure 6 gives the contribution analysis of life cycle stages (Fig. 6a) and materials and processes in the production stage (Fig. 6b). The contributions from different stages and processes do not vary much. S-III is shown as the example for the contribution analysis. It is found that the production stage contributes 61.35% to the single score and use stage contributes to 24.91% to the single score. EoL accounts for − 11.84%, while transportation stage has only 1.9% of the impact to the single score. The negative contribution of S-III in the EoL is caused by the recycling of waste steel wire and rubber recovery.

For the impact categories of CC, HOF, PMF, EOF and TA, the production stage is the largest contributor. The contributions from materials and processes to the production stage of these impact categories have similar distributions. CR, BR, steel wire and electricity consumption during the manufacturing are the primary contributors. Production of steel wire consumes large amount of coke and blast furnace gas. Butadiene in BR and calcium carbide in CR can lead to large environmental impacts. Electricity in China is generated 71.2% by thermal power, 16.4% by hydropower, 5.6% by wind energy, 4.9% by nuclear, and 1.9% by solar energy^47,48^. The impacts of electricity generation account about 10% of the impact of the production stage. The use stage is mainly dominated by fuel consumption. On the other hand, impacts of transportation and EoL are not significant in these impact categories.

In HTc, FET, MET and MRD, the production stage is the largest contributor, whereas large negative values are observed in the EoL stage. It is found that steel wire has the largest influence (58–77%) in the production stage, due to the consumption of hard coal in its upstream process. The use stage only accounts for 2.8–6.5% of the impacts. The EoL stage has − 34% to − 46% impacts, which is attributed to the high recycling rate of steel wires (97% recycled). Transportation has the lowest impact of less than 1%.

In WD and FRD, the use stage is the largest contributor, responsible for 88% and 67% of the impact, respectively. The large impact of the use stage in WD is 86% caused by water consumption in concrete pumping. On the other hand, fuel consumption is the primary contributor in the use stage for FRD, which accounts for 66% of the emission. In the production stage, CR is the largest contributor, which accounts for over 60% of the environmental impacts.

For the impact category of HTnc, Accelerator CZ and CR are the major contributors in the production stage. It is found that the impact of Accelerator CZ is 98%, caused by the adoption of cyclohexylamine. As the production of cyclohexylamine requires sulfidic tailing, of which the manufacturing process creates mercury and manganese, which are toxic to human health. In addition, the production of CR uses calcium carbide, which is dangerous due to its toxic effects.

### Results of life cycle costing (LCC)

The LCC results are provided in Table 11 and the calculated results have been validated with the factory manager. It is found that the production stage accounts for 41–45% of the life cycle cost, and the use stage accounts for 56–60% of the life cycle cost. On the other hand, the recovery of steel and waste rubber can lead to a profit of 3–3.5% of the life cycle cost. The transportation has only less than 2% of the life cycle cost. Among the four types of rubber hoses, S-III has the highest life cycle cost, followed by S-II, S-I and the Baseline. The production cost of S-III is 21% more than Baseline, which is mainly caused by the long life span of S-III (owing to the high resistant rubber of inner layer).

**Table 11:** LCC results of the four types of rubber hoses (FU: one rubber hose).

Stage	Item	Economic cost (¥)			
		Baseline	S-I	S-II	S-III
Production	NR	1.12E+02	1.21E+02	2.42E+02	1.61E+02
	CR	3.76E+02	3.76E+02	3.76E+02	3.76E+02
	SBR	6.67E+01	6.67E+01	6.67E+01	6.67E+01
	BR	3.39E+02	3.59E+02	2.43E+02	3.20E+02
	Sulfur	5.66E+00	5.97E+00	5.97E+00	5.97E+00
	Accelerator CZ	6.98E+00	0.00E+00	1.13E+01	7.54E+00
	Accelerator DM	6.31E+00	6.52E+00	3.64E+00	6.52E+00
	Accelerator NS	4.83E+00	1.43E+01	4.83E+00	4.83E+00
	Zinc oxide	5.42E+01	5.66E+01	5.66E+01	5.66E+01
	Stearic acid	5.01E+00	5.23E+00	5.23E+00	5.23E+00
	Carbon black	1.81E+02	1.91E+02	1.91E+02	1.91E+02
	Tackifying resin	3.79E+01	4.02E+01	4.02E+01	4.02E+01
	Antiager	2.10E+01	2.20E+01	2.20E+01	2.20E+01
	Paraffin	3.24E+00	3.43E+00	3.43E+00	3.43E+00
	Anti-scorching agent	2.66E−01	2.66E−01	2.66E−01	2.66E−01
	Magnesium oxide	4.57E+00	4.57E+00	4.57E+00	4.57E+00
	Adhesive	2.60E+00	2.60E+00	2.60E+00	2.60E+00
	Silica	2.81E+01	9.73E+00	9.73E+00	9.73E+00
	Silane coupling agent	4.61E+01	1.31E+01	1.31E+01	1.31E+01
	Aromatic oil	8.73E+00	8.73E+00	8.73E+00	8.73E+00
	Steel wire	2.22E+02	2.22E+02	2.22E+02	2.22E+02
	Electricity (Ningbo)	4.39E+01	4.39E+01	4.39E+01	4.45E+01
	Total (production)	1.58E+03	1.57E+03	1.58E+03	1.57E+03
Transportation	Transport (Ningbo to Qingdao)	6.90E+01	6.90E+01	6.90E+01	6.90E+01
Use stage	Water	1.54E+02	1.70E+02	1.81E+02	2.12E+02
	Diesel	1.80E+03	1.98E+03	2.11E+03	2.48E+03
	Total (use)	1.95E+03	2.15E+03	2.29E+03	2.69E+03
End-of-life	Waste delivery	1.10E+00	1.10E+00	1.10E+00	1.10E+00
	Reclaimed rubber	− 4.68E+01	− 4.68E+01	− 4.68E+01	− 4.68E+01
	Waste steel wire	− 1.57E+02	− 1.57E+02	− 1.57E+02	− 1.57E+02
	Electricity (Qingdao)	7.69E+01	7.69E+01	7.69E+01	7.69E+01
	Diesel	7.31E+00	7.31E+00	7.31E+00	7.31E+00
	Total (EoL)	− 1.18E+02	− 1.18E+02	− 1.18E+02	− 1.18E+02
Total		3.49E+03	3.69E+03	3.83E+03	4.23E+03

“¥” is Chinese Yuan (CNY).

### Results of the total costs

**Figure 7:**
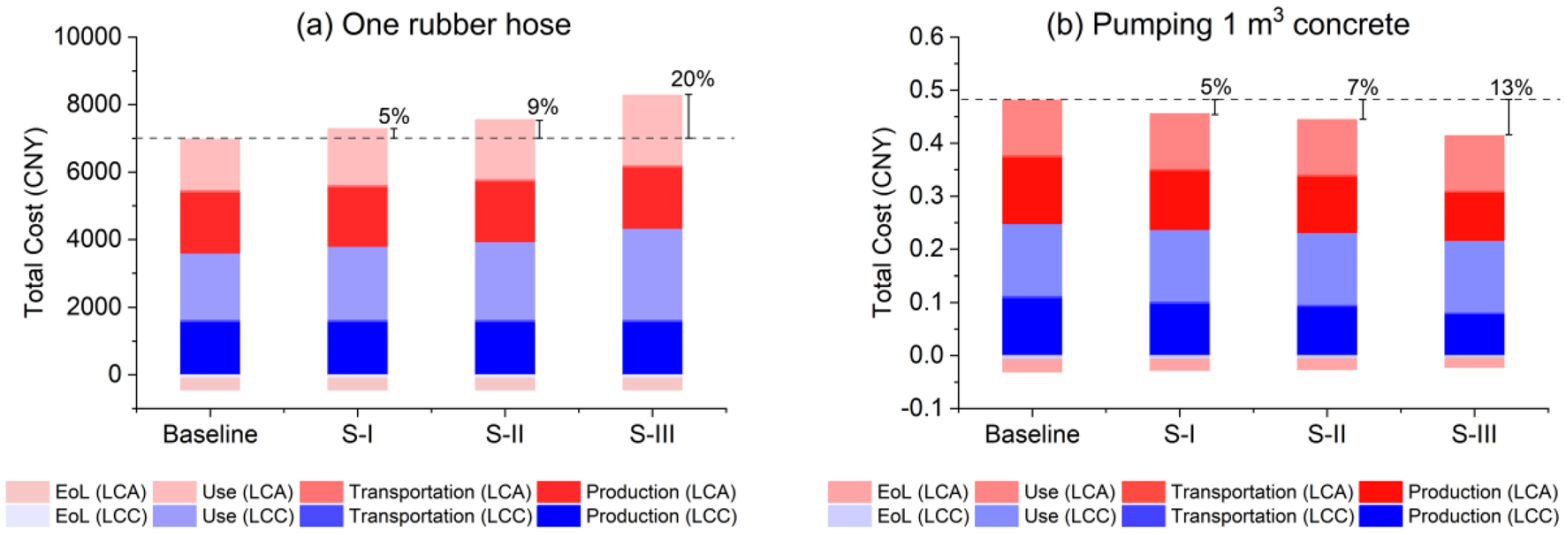
Integrated analysis of total costs of the four types of rubber hoses with different functional units: (**a**) one rubber hose and (**b**) 1 m^3^ concrete pumping.

The total costs of the four types of rubber hoses are shown in Fig. 7. It is found that the total cost of the Baseline rubber hose is 6528 CNY, in which the environmental costs account for 54% and the life cycle costs account for 46%. The profit due to the EoL recycling and recovery is 344 CNY. As compared to the Baseline, the total costs increase by 5%, 9% and 20% for S-I, S-II and S-III, respectively. However, when 1 m^3^ concrete pumping is chosen as the functional unit, it is found that S-III becomes the lowest costing rubber hose. The total cost of S-III to pump 1 m^3^ of concrete is only 0.39 CNY, while Baseline costs 0.45 CNY, which is 13% larger than S-III. Comparing S-III with Baseline, the environmental cost can be saved by 12% and the life cycle cost can be saved by 14%. To this regard, the selection of functional unit can largely determine the interpretation of the results. When the functional unit is 1 m^3^ concrete pumping, the high-resistance rubber hose S-III is the optimal choice with both low life cycle cost and low environmental cost.

## Discussion

### Sensitivity analysis

#### Monetary conversion factors

**Figure 8:**
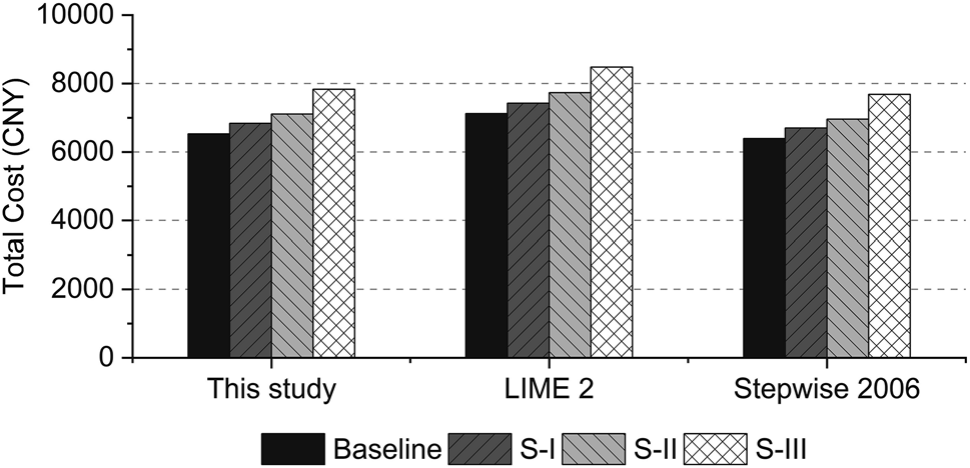
Total costs of the four rubber hoses calculated by different monetary valuation methods (FU: one rubber hose).

The conversion factors of environmental impacts can influence the total cost. In order to interpret the influence from the conversion factors, sensitivity analysis is carried out. The monetary valuation methods of LIME 2 and Stepwise 2006 are compared with the method adopted in this study. The conversion factors of this study, LIME 2 and Stepwise 2006 are shown in Table 12. The results of this sensitivity analysis are given in Fig. 8. It is found that the results from LIME 2 are 8–9% higher than the results of this study. On the contrary, the results from Stepwise 2006 are about 2% lower than this study. Moreover, the ranking of the damage categories is consistent in the three methods, with human health, ecosystems and resources from the highest to the lowest. With respect to the four types of rubber hoses, the Baseline has the lowest total cost, followed by S-I and S-II, whereas S-III has the highest total cost.

**Table 12:** Endpoint monetary conversion factors of different monetary valuation methods.

Method	Human health		Ecosystems	
	Value	Unit	Value	Unit
This study	100,000	USD/DALY	65,000	USD/species.yr
LIME 2	142,623	USD/DALY	13,775	USD/species.yr
Stepwise 2006	90,206	USD/DALY	3755	USD/species.yr

The values of conversion factors are normalized to the unit of USD/DALY and USD/species.yr. The exchange rate is 1 EUR = 1.219 USD^44^; 1 DALY = 1 QALY, Quality Adjusted Life Year^49^; 1 species.yr = 10,000,000 species^50^.

#### Materials and processes in the production stage

Adjusting the composites in the rubber hoses can lead to different environmental performances. In order to identify the impacts caused by changing ingredients in rubber composites and detect the hotspots of emissions, sensitivity analysis on the materials and processes in the production stage is conducted. We selected the adjustment of ingredients according to the practice of laboratory tests. The ingredients are adjusted by 5%, 10%, 15% and 20%. The sensitivity analysis is conducted for individual impact categories, environmental single score, and the total cost.

##### Individual impact categories

**Figure 9:**
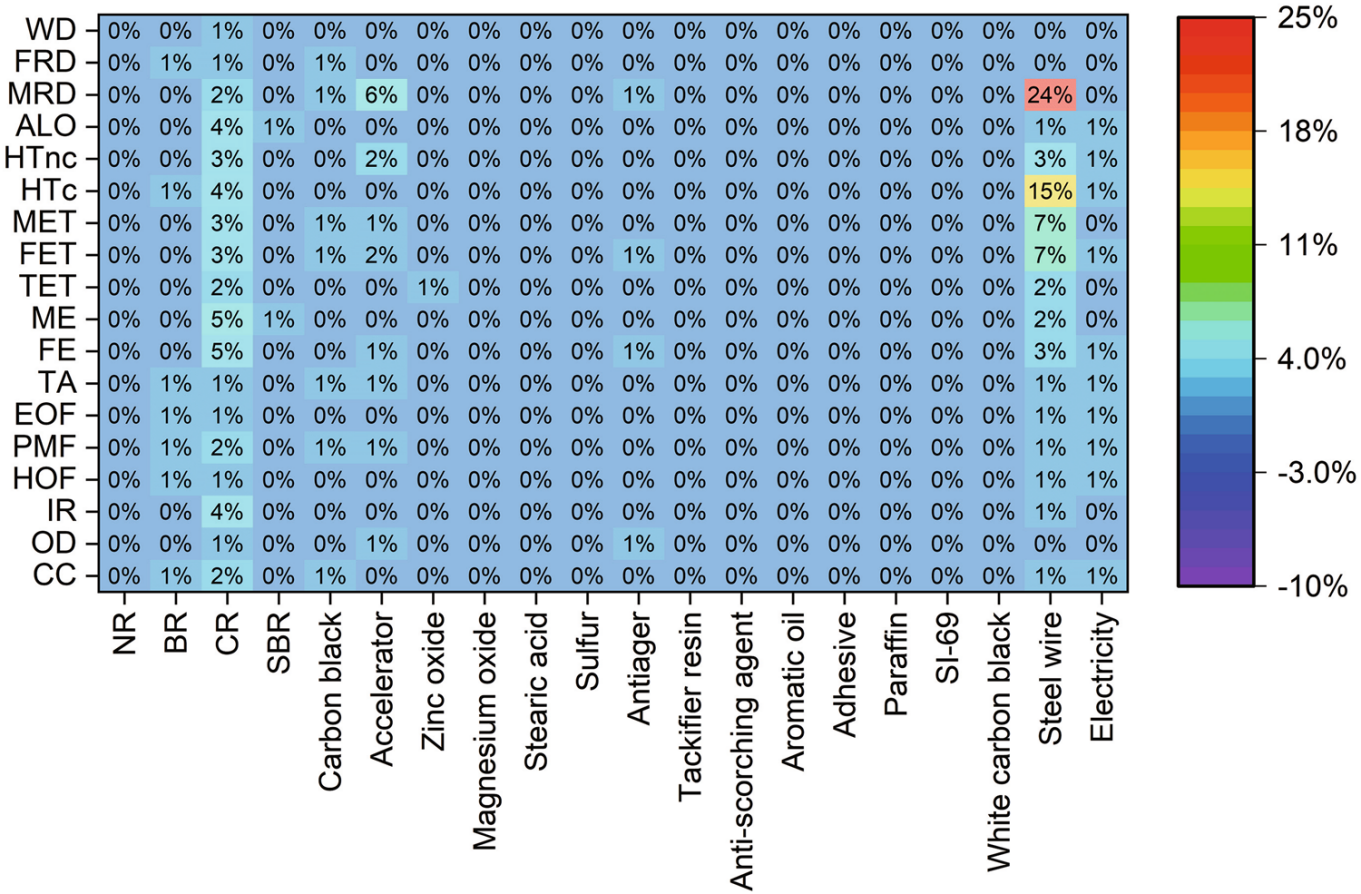
Changes of the midpoint results of the entire life cycle of S-III caused by 10% increase of the materials and processes in the production stage.

Sensitivity analysis is conducted for the materials and processes in the production stage of S-III. By increasing 10% of inputs in the production stage, the changes of midpoint results for the entire life cycle of rubber hoses are analyzed. As shown in Fig. 9, it is found that most of the inputs have only less than 1% influence, except for CR, Accelerator, and Steel wire. When CR is increased by 10%, 5% growth is observed in FE and ME, followed by 4% growth in ALO, HTc and IR. With 10% increase of accelerators, the results of MRD are increased by 6%, followed by 2% growth in HTnc and 2% growth in FET. The most influential material in the production stage is steel wire. It is found that 10% increase of steel wire can result in a growth of MRD by 24%, HTc by 15%, and MET and FET by 7%. On the other hand, the impact categories of WD, FRD, TA, EOF, HOF, and OD can be slightly affected by the materials or processes in the production stage.

##### Environmental single score

**Figure 10:**
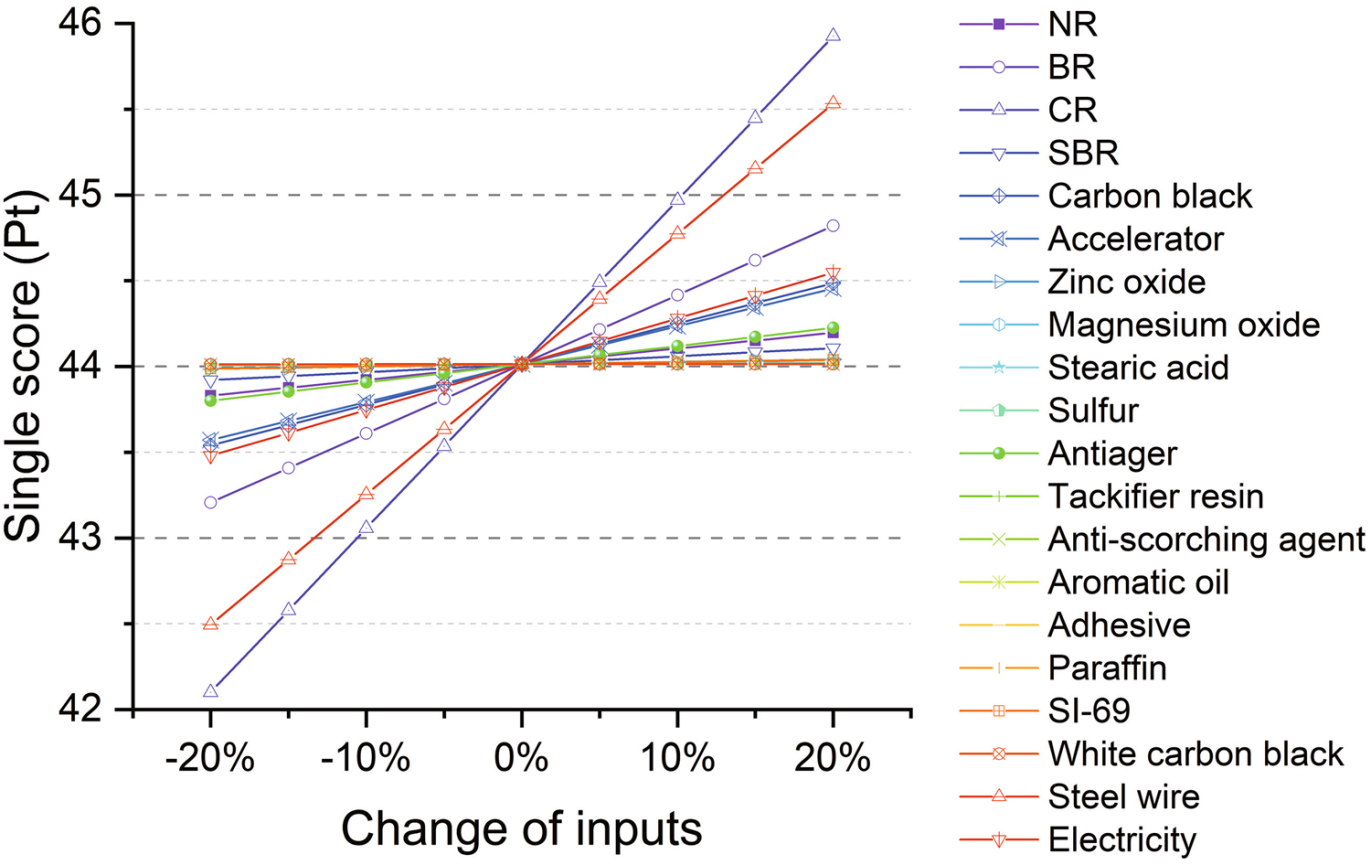
Sensitivity analysis of inputs in the production stage of S-III to the single score.

The influences from materials and processes to the single score are evaluated through a sensitivity analysis. In Fig. 10, all the inputs are changed by − 20%, − 15%, − 10%, − 5%, 0%, 5%, 10%, 15% and 20%. The influence is linear for all the materials and processes. The linearity of LCA has been observed in most LCA studies, whereas non-linearity of LCA may take place for some special cases, such as in a cradle-to-cradle scope (the inputs are non-linear)^51^. CR is found to be the most influential material to the single score. 10% increase of CR can result in 2.17% increase of the single score. Steel wire is the second most influential material, 1.73% growth of which is observed with 10% increase in the input. Following CR and steel wire, other important materials/processes are BR, electricity consumption, carbon black, and accelerators. The remaining materials/processes have only less than 0.5% of the influence to the single score with 10% increase of the inputs.

##### The total cost

**Figure 11:**
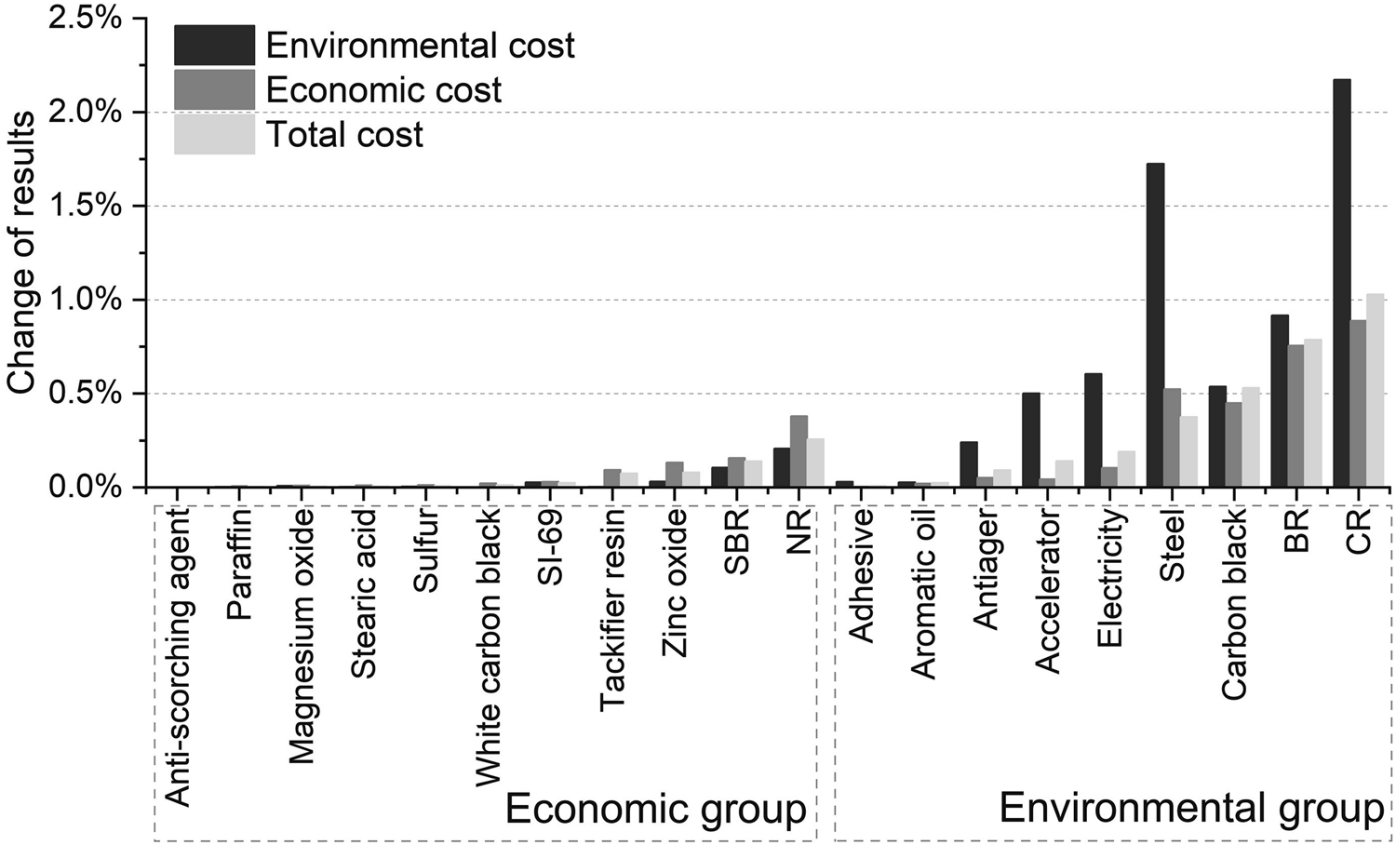
Sensitivity analysis of inputs in the production stage of S-III to environmental cost, economic cost and total cost (with 10% increase of the input).

Figure 11 gives the influence from inputs in the production stage to the environmental cost, economic cost and total cost. It is found that the total cost is most sensitive to CR. With 10% increase of CR, the total cost is increased by 1.03%. BR is the second influential input and 10% increase of BR can lead to 0.79% increase of the total cost. Carbon black is the third influential input and the total cost has 0.53% growth when carbon black input is increased by 10%. On the other hand, the total cost is not sensitive to anti-scorching agent, paraffin, magnesium oxide, stearic acid, sulfur, and adhesive, with only less than 0.1% change for the increase of inputs by 10%.

The environmental cost is more sensitive to CR, steel wire, BR, electricity, carbon black, accelerator, and antiager (the environmental group), whereas the economic cost is less sensitive to these inputs. For example, by increasing 10% CR, the environmental cost is increased by 2.2% while the economic cost is increased by 0.89%. In contrast, the environmental cost is less sensitive to NR, SBR, zinc oxide, tackifier, etc. (the economic group), while these inputs are more influential to the economic cost. Therefore, when the rubber composites are re-designed for the purpose of environment protection, the materials or processes in the environmental group should be considered.

#### Influence from resources

**Figure 12:**
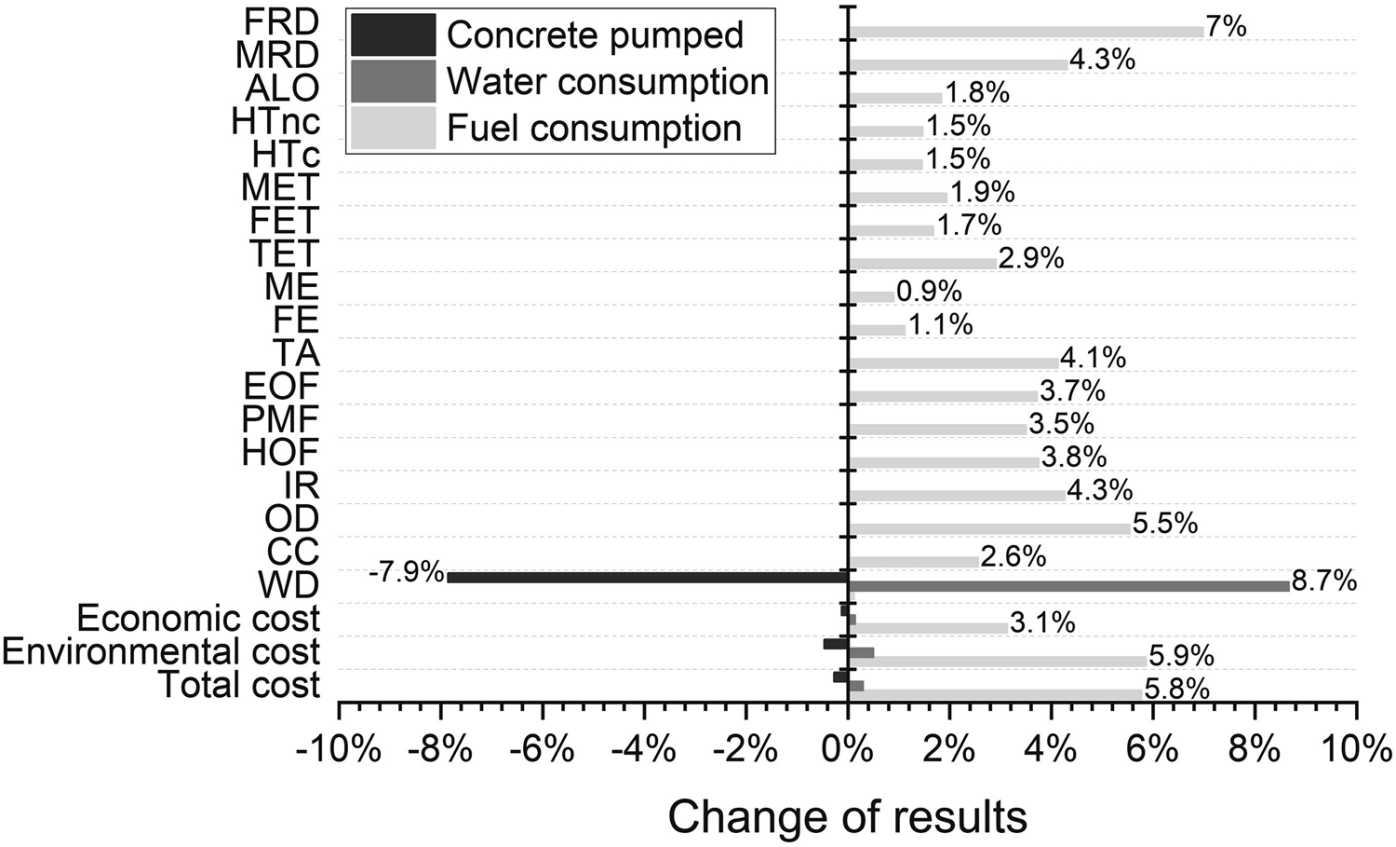
Sensitivity analysis of water consumption, fuel consumption and the volume of concrete pumping between two flushes (the results of S-III are presented).

In the use stage, water consumption and fuel consumption are found to be the main contributors to the environmental single score and life cycle cost. In order to interpret the impacts of these two inputs, sensitivity analysis is conducted with an increase 10% of water consumption and fuel consumption as inputs. In addition, the influence caused by the concrete volume pumped between two flushes is investigated by increasing the 200 m^3^ concrete to 220 m^3^ (10% increase). The results of sensitivity analysis are shown in Fig. 12. It is found that the increase of concrete volume as pumped between flushes can largely reduce the impact to water depletion, and save the total cost. However, the increase of water consumption by 10% can lead to 8.7% of the impacts to WD. In addition, the fuel consumption can significantly influence FRD, OD, TA, IR, MRD, etc. By increasing 10% of the fuel consumption during the use stage, the total cost is increased by 5.8%.

### Comparison with previous studies

In order to validate this study, the midpoint impact of CC in the production stage is compared with the previous studies. Since there has been no similar study on rubber hoses, we compared the result with other rubber products, e.g., a passenger car tire, an electric bicycle tire, and glove. As shown in Table 13, the results of rubber hoses of this study are comparable to the results of tires in previous studies. On the other hand, the GHG emission of glove is apparently lower than that of rubber hoses or tires. The structure as well as the composite of rubber hoses is analogue to tires, but different significantly from gloves. Gloves are mainly made of rubber with over 89% of weight^52^, whereas rubber hoses are composed of rubber (37%), steel wire (37%), carbon black (17%), etc. Therefore, the GHG emissions of rubber hoses in this study are apparently higher than gloves.

**Table 13:** Comparison with previous studies on CC for the production stage.

Studies	^42^	^53^	^52^	This study
Object	Passenger car tire	Electric bicycle tire	Glove	Rubber hose (S-III)
CC (kg CO_2_ eq/kg product)	4.38	3.78	1.88	4.04

### Recommendations

The production of rubber hoses contributes significantly to the life cycle environmental impacts. The key contributors in the production stage are CR, BR, steel wire and electricity consumption. As a result, future work is recommended to study the alternative designs which can reduce the amount of high-impact ingredients and manufacturing processes with decreased electricity consumption. Accelerator CZ is found to be influential to EF, ME, FET, MET, HTnc and MRD, thus it is necessary to control the dose of Accelerator CZ in rubber hose production. During the use stage, fuel consumption is the primary contributor to all the environmental impact categories. As a result, it is necessary to reduce the fuel consumption to reduce the environmental impacts during the use stage. As a large amount of water is consumed for pipe cleaning, the increase of the concrete pumping volume between flushes can considerably reduce water consumption. The recycling of waste rubber and steel wire is strongly suggested, as this can benefit on both the environmental and economic aspects.

When the economic cost is taken into account, the items with high environmental impacts are however not influential to the economic cost. According to the sensitivity analysis, the items can be classified into economic group and environmental group. The economic group has larger economic impacts but lower environmental impacts, vice versa. When improving the rubber hose design, it is necessary to consider both the environmental and economic impacts, rather than just focusing on one aspect of impacts. It is strongly recommended to calculate the total cost that embraces both environmental and economic aspects by applying the method developed in this study. This shall prevent a biased selection of rubber hose design and facilitate the scientific eco-design of rubber hoses as well as other rubber products.

## Conclusions

This study analyzes the environmental and economic performance of rubber hoses by developing a multi-objective optimization method that integrates life cycle assessment (LCA) and life cycle costing (LCC). Four types of rubber hoses with inner rubber layer of different composites are compared and a series of sensitivity analyses are conducted. S-III is the high wear resistant rubber hose that can pump 20,000 m^3^ concrete. It is found that, when the functional unit (FU) is one rubber hose, S-III has the largest greenhouse gas (GHG) emission of 851 kg CO_2_ eq, while S-II has the lowest emission of 793 kg CO_2_ eq. The single score of S-III is also the largest among the four rubber hoses. The economic cost and the total cost (economic and environmental costs) of S-III are the highest, while Baseline is the lowest. However, when the FU is set as 1 m^3^ concrete pumping, S-III has the lowest environmental single score, life cycle cost, and total cost, respectively among the four rubber hoses. S-III can save 13% total cost comparing to the Baseline, indicating the high wear resistant rubber hose is both environmentally and economically optimized.

The contributions to the environmental single score are 61% by production, 25% by use, 2% by transportation and − 12% by EoL. On the other hand, life cycle cost is mostly contributed from use (56–60%), but less contributed from production (41–45%). In the production stage, CR, BR, steel wire and electricity are the primary contributors to the environmental impacts, whereas the economic cost is not sensitive to these items. Fuel consumption in the use stage is influential to both environmental and economic impacts, hence the reduction of fuel consumption in the concrete pumping is of paramount importance. The total cost is 54% contributed from environmental cost and 46% from economic cost.

The results from this study are verified by comparing with the previous studies. It is recommended to reduce CR, BR, steel wire, and electricity consumption in the production stage, and water and fuel consumption in the use stage to improve the environmental performance. In addition, improving the efficiency in concrete pumping by increasing the pumping volume between flush intervals can benefit both environmental and economic aspects. The integrated method as developed in this study facilitates the eco-design of rubber hoses used in construction sites and can be implemented in other rubber products.

## Supplementary Information

Supplementary Information.
