# Supplementary material for: Integration of life cycle assessment and life cycle costing for the eco-design of rubber products
Source: Sci Rep. 2022 Jan 12;12:595. doi: 10.1038/s41598-021-04633-6 (PMC8755712; doi:10.1038/s41598-021-04633-6)
Supplement: Supplementary file 1 — Supplementary Information. [file 41598_2021_4633_MOESM1_ESM.pdf]

Questionnaire Survey

on

Life Cycle Assessment of Rubber Hoses

Name of Participant: ___________ Location: ____________ Date: ____________

1. General information

1.1 Products

Product：	
Type：	
Function：	
Made in：	
Application：	
Length：	
Inner diameter：	
Outer diameter：	
Weight of one hose:	
Functional unit*：	

*The following data should be normalized to the function unit.

1.2 Properties

DIN Abrasion： ____________________； Tensile strength： _______________________；

Tearing strength： ________________； Elongation at break： ____________________；

2.Inputs and emissions

2.1Raw material

Material	Type	Amount (per FU)	Unit	Origin	Proportion of materials*
Natural rubber					
Synthetic rubber					
Carbon black					
Stearic acid					
Paraffin					
Zinc oxide					
Sulfur					

* Inner Rubber: I; Outer rubber: O； Reinforcement: M； I-30% refers to 30% of the material is used in inner rubber.

2.2 Energy and resources

Item	Amount (per FU)	Unit	Proportion*
Electricity			
Natural gas			
Coal			
Diesel			
Petroleum			
Water			

*Proportion of certain process in energy/water consumption.

3. Transportation (from manufacturer to user)

Mode	Fuel	Distance（km）
Truck*（____）		
Truck*（____）		
Truck*（____）		
Truck*（____）		
Rail		

*Please fill in the truck type.

4. Use stage

Life span：_______________ （□Month/□Year）

Concrete pumped during life span：_______________ （□kg/□t/□m^3）

5. End-of-life stage

Is the waste hose recycled, reused or recovered? □Yes □No

If "Yes"， please specify the method (e.g., incineration): ____________________

6. Others

Please provide the following documents (if available):

□Flow chart of manufacturing □Photos of manufacturing

□Photos of the products □Photos of product implementation

Thank you for your support!
